# Preventing relapse with personalized smart‐messaging after cognitive behavioural therapy: A proof‐of‐concept evaluation

**DOI:** 10.1111/bjc.12244

**Published:** 2020-01-20

**Authors:** Sam Malins, Sanchia Biswas, Shireen Patel, Jo Levene, Nima Moghaddam, Richard Morriss

**Affiliations:** ^1^ University of Nottingham UK; ^2^ Nottinghamshire Healthcare NHS Foundation Trust UK; ^3^ University of Lincoln UK

**Keywords:** cognitive behavioural therapy, digital health interventions, relapse prevention, telehealth

## Abstract

**Objectives:**

Cognitive behavioural therapy (CBT) can improve symptoms of anxiety and depression, but also reduces the risk of future relapse after therapy completion. However, current CBT relapse prevention methods are resource‐intensive and can be limited in clinical practice. This paper investigates a personalized means of reducing relapse using smart‐messaging in two settings: research and routine care.

**Design:**

Study 1 presents a cohort study comparing a cohort of smart‐messaging users versus non‐users. Study 2 presents time series follow‐up data from a case series of smart‐messaging users from clinical practice.

**Methods:**

Fifteen of 56 CBT completers who participated in a trial for the treatment of health anxiety wrote advice they would want if in future they were doing well, experiencing early warning signs of relapse, or experiencing full relapse. Following CBT, participants received weekly text‐message requests to rate their well‐being. Dependent upon their response, participants received tailored advice they had written, appropriate to the well‐being level reported after recovery from health anxiety. Smart‐messaging was also trialled in a routine practice sample of 14 CBT completers with anxiety and depression.

**Results:**

Across a 12‐month follow‐up, participants receiving smart‐messaging showed greater health improvements than those who did not. Well‐being scores showed stability between CBT completion and 6‐month follow‐up among routine care patients.

**Conclusions:**

These findings suggest that a low‐intensity, personalized relapse prevention method can have a clinical benefit following CBT for common mental health problems.

**Practitioner points:**

- Post‐treatment outcomes may be improved using personalized smart‐messaging to prevent relapse following cognitive behavioural therapy (CBT) for health anxiety.
- In clinical practice, post‐treatment smart‐messaging can be well‐used by patients and may help maintain stable well‐being in the 6 months after CBT ends.
- This evidence supports the clinical utility of a brief tailored digital intervention, which can be integrated within routine clinical practice with minimal therapist input.
- Overall, longer‐term post‐CBT outcomes may be improved by integrating a smart‐messaging intervention at the end of therapy.

## Background

Anxiety and depressive disorders are common in the general population and are often recurrent with a chronic course (Kessler, Chiu, Demler, & Walters, 2005; Penninx *et al.*, 2011; Steel *et al.*, 2014). Because of this, there are long‐term implications for such disorders including early retirement and comorbid chronic physical complaints (Hendriks *et al.*, 2015; Wittchen *et al.*, 2011). Most people with anxiety and depression experience a lower level of functioning and residual symptoms of recurrence even if recovery is achieved (Rhebergen *et al.*, 2011). Effective treatments, such as antidepressant medication, are available, but relapse rates remain high after discontinuation of treatment (Scholten, Batelaan, Van Oppen, Smit, & Van Balkom, 2013). Cognitive behavioural therapy (CBT) is effective in improving symptoms of anxiety and depressive disorders, and post‐treatment relapse rates can be lower than those achieved by antidepressant medication alone (Cuijpers *et al.*, 2013; Hollon *et al.*, 2005).

The post‐treatment relapse prevention attributes of CBT have been linked to the skills learnt by patients attending CBT, because patients treated with CBT experienced more change in thoughts and beliefs than those treated with antidepressants (DeRubeis *et al.*, 1990; DeRubeis, Siegle, & Hollon, 2008). Furthermore, CBT patients independently rated as acquiring specific CBT skills and competencies through therapy were at lower risk of relapse after treatment than those who did not, even after controlling for post‐treatment severity and change over treatment (Strunk, DeRubeis, Chiu, & Alvarez, 2007). These studies suggest that acquisition of CBT‐related coping skills is an important component of relapse prevention. However, 30–50% of patients attending CBT for anxiety and depressive disorders still relapse within the first year after treatment, with greater relapse rates found in clinical practice than controlled research conditions (Delgadillo *et al.*, 2018; Hollon *et al.*, 2005).

To address the substantial problem of relapse even after CBT for anxiety and depressive disorders, a number of therapy enhancements have been developed to prevent post‐CBT relapse. These interventions have typically involved additional therapy sessions focused on developing relapse prevention plans, identifying potential triggers for relapse, and learning coping strategies to manage such eventualities (Bockting *et al.*, 2005). Many of these interventions are included in longer CBT treatment protocols, but CBT is often shorter in clinical practice with abbreviated relapse prevention work (Hansen, Lambert, & Forman, 2002; NHS Digital, 2018). Attenuating relapse prevention activities may reduce the long‐term effectiveness of CBT interventions, with 53% of patients relapsing within 1 year from brief, low‐intensity CBT‐type interventions for anxiety and depression (Delgadillo *et al.*, 2018). This is considerably higher than relapse rates following CBT including relapse prevention content (39%; Vittengl, Clark, Dunn, & Jarrett, 2007). Overall, this suggests that relapse prevention interventions may have a benefit in helping patients maintain improvements achieved in therapy.

However, CBT interventions to specifically prevent relapse are time‐ and resource‐intensive. Therefore, a number of alternative, digital means of reducing relapse have been developed in recent years, which have several benefits. Digital health interventions may help address the resource‐ and time‐related barriers preventing routine use of interventions to reduce relapse in clinical practice, because of their low cost and ease of access (Lingam & Scott, 2002; Paganini, Teigelkoetter, Buntrock, & Baumeister, 2018). Digital health interventions (i.e., interventions delivered via desktop computers, tablets, or mobile phones, often using Internet‐based resources) can achieve similar effects to high‐intensity traditional approaches, but using much less therapist time (Hennemann, Farnsteiner, & Sander, 2018). Digital health interventions have been used to increase the reach and accessibility of relapse prevention interventions in several recent trials. Meta‐analyses of digital relapse prevention interventions suggest they can be effective for anxiety and depressive disorders, but the benefits are moderated by the degree of personalization included (Hennemann *et al.*, 2018). Furthermore, minimal, but personalized, relapse prevention methods that include an individual plan are preferred if regular contact with a mental health professional is not an option (Muntingh *et al.*, 2019). This suggests digital tools may present an effective and efficient means of reducing relapse in anxiety and depressive disorders.

Conversely, there are identified weaknesses in digital health interventions. Specifically, low‐intensity, automated interventions have historically had weaker effects than high‐intensity interventions delivered face to face (Rodgers *et al.*, 2012). Furthermore, few digital relapse prevention programmes are integrated with traditional one‐to‐one CBT, despite this remaining the treatment medium most likely to be chosen by patients (Berle *et al.*, 2015). Therefore, current digital relapse prevention methods are often unable to harness the knowledge and learning gained by patients during CBT that can be key in preventing relapse (Strunk *et al.*, 2007).

Current evidence suggests that brief, easily implemented reminders, such as targeted text messages, can act as effective prompts for therapeutic and healthy behaviour change (Boksmati, Butler‐Henderson, Anderson, & Sahama, 2016; Hall, Cole‐Lewis, & Bernhardt, 2015; Robotham, Satkunanathan, Reynolds, Stahl, & Wykes, 2016). ‘Smart‐messaging’ interventions like this can improve physical and mental health across a range of health problems (Rathbone & Prescott, 2017). Yet, smart‐messaging has not been used, in a personalized way, to enhance relapse prevention in CBT for anxiety and depressive disorders, despite evidence that smart‐messaging is one of the easiest and most cost‐effective digital interventions to integrate within routine care (Boksmati *et al.*, 2016; Hall *et al.*, 2015; Wong *et al.*, 2016). Using personalized smart‐messaging prepared by the therapist and patient before CBT ends may help to draw on the strengths of digital health interventions and overcome the difficulties associated with low‐intensity interventions.

This study evaluates initial evidence of effectiveness and feasibility for a personalized, minimal contact, post‐CBT digital intervention that is integrated with key learning from therapy. It applies well‐used smart‐messaging technology (The King's Fund, 2018) to regularly deliver personalized relapse prevention messages written by the individual patient and tailored to the patient’s current mood state. This intervention addresses a key research gap, as no studies have investigated integrated, minimal contact, personalized smart‐messaging interventions for relapse prevention post‐CBT in anxiety and depressive disorders (Hennemann *et al.*, 2018).

This paper is separated into two observational studies to evaluate the feasibility and clinical effectiveness of smart‐messaging as a post‐CBT relapse prevention tool: Study 1 compares long‐term clinical outcomes of smart‐messaging relapse prevention users with non‐users from a clinical trial sample of patients with health anxiety and multiple comorbidities. Study 2 assesses the feasibility of smart‐messaging use in routine clinical practice with cancer patients experiencing comorbid mental health problems. Each study is described in turn, and a joint discussion is presented at the end.

Overall, given the recent rise in stand‐alone digital health interventions, this paper presents proof‐of‐concept evidence for a digital health intervention which emphasizes the personalized approaches associated with greater outcome improvement. The intervention also closely integrates with one‐to‐one CBT processes, which remains the dominant means of CBT provision.

## STUDY 1: CLINICAL OUTCOMES OF SMART‐MESSAGING USERS VERSUS NON‐USERS FOLLOWING REMOTELY DELIVERED CBT FOR HEALTH ANXIETY

## Method

### Design and setting

An exploratory observational design was applied to data from a randomized controlled trial of remotely delivered CBT for health anxiety versus usual care among high service utilizers (Morriss *et al.*, 2019). The 791There were 78 participants randomized to CBT, and one participant was randomized to usual care but offered CBT in error. Their data are included in the analysis. Therefore, the total sample is 79. participants randomized to CBT were offered 5–15 sessions delivered via either videoconferencing (*n* = 54) or the telephone (*n* = 14); 11 did not attend any sessions. The current study’s objective was to compare clinical outcomes from participants who completed treatment and used post‐treatment smart‐messaging with treatment completers who did not use post‐treatment messaging.

### Participants

Participants were recruited from primary and secondary care after being approached by their treating clinician. If participants scored ≥18 on the 14‐item Short Health Anxiety Inventory (SHAI) and attended two or more urgent care appointments in the previous 12 months, they were eligible for inclusion. Participants were excluded if they were at immediate risk of harm to themselves or others and had moderate‐to‐severe intellectual disability or severe mental or physical illness to the extent that engagement in the intervention would not be possible (e.g., communication difficulties). Fifty‐four patients were excluded from the source trial due to not meeting eligibility criteria.

Fifty‐three of the 79 participants were classified as ‘completers’ (attended at least five sessions), which is deemed an adequate treatment dose for clinical effect in CBT for health anxiety (Tyrer *et al.*, 2014). Fifteen CBT completers opted to trial post‐treatment smart‐messaging (smart‐messaging group), and 38 did not (no‐messaging group). Smart‐messaging participants attended a mean of 11.9 (*SD* = 2.4) sessions, and no‐messaging participants attended 11.1 (*SD* = 3.0). At 6 months, 100% follow‐up was achieved for smart‐messaging users and 84% for non‐users. At 12‐month follow‐up, 93% follow‐up was achieved for smart‐message users and 68% for non‐users.

### Interventions

#### CBT for health anxiety

An established CBT for health anxiety treatment protocol was adapted for remote delivery through collaboration between two patient advisors and a CBT therapist (Patel *et al.*, 2016; Tyrer *et al.*, 2014; Tyrer *et al.*, 2011). Treatment included identification of key beliefs and assumptions about health and illness, followed by testing and evaluation of beliefs using behavioural experiments. Potentially problematic anxiety‐maintaining cognitive and behavioural strategies, such as repeated reassurance‐seeking or body checking, were collaboratively identified and reduced or stopped.

#### Post‐treatment smart‐messaging

An established smart‐messaging system was used to develop the post‐treatment messaging intervention (http://www.simple.uk.net/). Their smart‐messaging system is employed across several different health services to help people manage their health long term (The King's Fund, 2018).

In the final two CBT sessions, a relapse prevention plan was developed. Participants were asked if they wished to have elements of their relapse prevention plan sent to them in text messages after CBT sessions had finished. Those who gave written consent to this were then led through the following process: Using template worksheets (see Supporting information), participants and therapists firstly identified characteristic patterns of responding for the individual patient when: (1) doing well, (2) experiencing early warning signs of relapse, and (3) experiencing full relapse. These included patterns of thought, behaviour, and characteristic emotional responses. Secondly, the participant was asked to imagine themselves in 3–6 months’ time: (1) doing well, (2) experiencing early warning signs of relapse, and (3) experiencing full relapse. The participant was then asked what advice or actions they would suggest if they were able to send themselves a text message under each of these three circumstances. The patient and therapist then collaboratively developed a series of brief advice messages for each of the three levels using the learning gained from CBT sessions (see Table 1 for example messages for each level). Finally, participants were asked if they were able to come up with a slogan or headline summarizing what they had learnt from therapy that they felt would help them stay well in future. Therapists encouraged patients to make these slogans as personally relevant as possible, rather than general statements. For example, one participant wrote the message ‘Uncompleted projects are not necessarily failures’ as a summary of key learning from therapy. This is not necessarily a key issue for people with health anxiety in general, but for the participant in question this was a focus of therapeutic work; much anxiety had revolved around self‐criticism for not finishing projects. Participants were encouraged to write three or more summary headlines (Table 2 gives example headline summaries written by participants).

**Table 1:** Example relapse prevention messages

Relapse stage	Personal experience	Personal advice message
Doing well	Enjoy planning my days [ ] look forward to events, meets with friends etc.	Be proud of yourself for the things you are achieving and doing. [P 03014]
	Going to the gym, playing football and with kids. Getting on well with family and friends. In the moment, enjoying life	You’ve got a good plan in place – stick to it. Sleep, relax, eat well, reduce your stress! [P 01031]
Early warning signs of relapse	Becoming more preoccupied with health problems. Probably start looking up symptoms on the [inter]net which in turn will make my anxiety worse	Try to remember that looking on the [inter]net can give false information and may sensationalise symptoms. Learn by your mistakes. Try and avoid constantly asking for reassurance. [P 01046]
	Feeling less able to control my worrying and may start to effect work, start making excuses not to see friends/family, not exercis[ing] as much	Try and do things that you enjoy doing, like going running even if you don’t feel like it. Be the yoga me! [P 03009]
Full relapse	I start thinking that my friends actually don’t like me and that everyone criticises me behind my back. Overthink	Your friends like you, otherwise they would not hang out with you. You are loved [P 01104]
	Pacing about, needing reassurance, feeling panicky, tingling in hands and feet, [want] to see [the family doctor]	This is anxiety bullying you, there is nothing else wrong. Tell your anxiety bully where to go. [P 06005]

**Table 2:** Headline summary message examples

Age range	Gender	Personal headline reminder message
50–60	Female	The fear will pass. I can cope, I have coped [P 01071]
20–30	Male	Generally anxieties come and go, often they resolve on their own. [P 01084]
40–50	Female	Keep in touch with family and friends [ ]. They won’t think any less of you even if you don’t feel 100% and seeing people makes you feel better. [P 01024]
20–30	Female	You are not your thoughts and they do not control or define you! [P 01108]
20–30	Female	Do things for fun, even in stressful times, plan in leisure time (cooking, sewing) [P 01096]
30–40	Female	[Try] not to look to Google or check my body for answers – it doesn’t help! [P 02007]
60–70	Male	Try to continue as if the anxiety were not there and act as you would usually. Don’t let the anxiety interrupt your plans! [P 02006]

For each of the 25 weeks after CBT sessions had finished, participants received a text message asking them to rate their well‐being that week from 0 to 5 in a responding text, where 0 was the worst possible well‐being and 5 was the best. If the participant responded with 5 or 4, they received a text message containing some of the advice they had written and wanted to receive if doing well. If participants responded with 3 or 2, they received some of their own advice for if they were experiencing early warning signs of relapse. If the participant responded with 1 or 0, they received their own advice for managing full relapse. At a different point each week, participants also received a message containing one of their summary slogans.

### Outcome measures

Outcomes were collected by independent researchers, blind to treatment group allocation at baseline, 3‐, 6‐, 9‐, and 12‐month follow‐up. Given the high rates of comorbidity among health anxiety sufferers, a range of physical and mental health outcomes was used to assess therapeutic change:

- The 14‐item Short Health Anxiety Inventory (SHAI) is a measure of health anxiety assessing worries about health and bodily changes. The SHAI shows excellent test–retest reliability (*r* = .90) and convergent validity with assessment of hypochondriasis (*r* = .85) (Salkovskis, Rimes, Warwick, & Clark, 2002).
- The seven‐item Generalised Anxiety Disorder (GAD‐7) Scale is a measure of generalized anxiety symptoms based on diagnostic criteria with excellent test–retest reliability (ICC = .83) and good convergent validity with other anxiety measures (*r*s = .72 to .74) (Spitzer, Kroenke, & Williams, 2006).
- The nine‐item Patient Health Questionnaire (PHQ‐9) measures depression symptoms based on diagnostic criteria for major depression. Excellent test–retest reliability has been demonstrated (α = .84) and good convergent validity with general mental health assessment (*r* = .73) (Kroenke, Spitzer, & Williams, 2001).
- The EuroQol – 5 Dimensions – 5 Levels (EQ‐5D‐5L, Herdman *et al.*, 2011) is a measure of quality of life assessing functional, physical, and mental health components of life quality. It has shown acceptable test–retest reliability (ICC = .52) and good convergent validity with established assessments of well‐being (*r_s_* = .77) (Janssen, Birnie, Haagsma, & Bonsel, 2008; Janssen *et al.*, 2013).
- The visual analogue scale (VAS, Herdman *et al.*, 2011) assesses general health, as part of the EQ‐5D, and shows similar psychometric properties.
- The 15‐item Patient Health Questionnaire (PHQ‐15) is a measure of somatic symptoms, wherein psychological distress is manifested in physical symptoms. Acceptable test‐retest reliability has been achieved (*r* = .56) (Kroenke, Spitzer, & Williams, 2002).
- The Work and Social Adjustment Scale (WSAS; Mundt, Marks, Shear, & Greist, 2002) assesses functional impairment and has shown good‐to‐excellent test–retest reliability (*α* = .73) and moderate‐to‐strong correlation with clinician interview (*α = *.81 to .86) and depressive symptomatology (*α* = .76). The focal problems identified for assessment on the WSAS were described as symptoms or pain (e.g., Because of my symptoms/pain, my ability to work is impaired).

### Procedure

If potential participants consented to contact, independent researchers then carried out a telephone screening for health anxiety severity and service use. Researchers conducted follow‐up assessments by telephone, videoconference, in person, or by email depending on participant preference (See Morriss *et al*, 2019 for more details).

### Method of analysis

The clinical and demographic characteristics of CBT completers who received smart‐messaging were compared to those who did not. Mann–Whitney U and chi‐squared tests were used to compare characteristics for continuous and categorical variables, respectively.

The outcomes in the smart‐messaging and no‐messaging groups were compared using multilevel modelling, given the repeated measurement design. A significant 62–78% of outcome covariance was explained by clustering within participants over follow‐up assessment time. This suggested that multilevel modelling was an appropriate analytic method to account for both within‐ and between‐participant variance over time. A two‐level hierarchical data structure was applied, nesting ordered assessment time points (‘time’ hereafter – level 1) within associated participants (level 2). This method also offered more accurate estimates of change over the 12‐month follow‐up period, whereas more typical multiple linear regression analyses usually rely on a single assessment time point and are more affected by missing data. Applying conventional model building guidelines, each parameter was added to the model individually and the goodness of fit was assessed using −2 log likelihood (Raudenbush & Bryk, 2002). Parameter estimation was made using maximum likelihood. Fully adjusted models included random intercepts and fixed slopes with level 2 predictor variables: *time* (adjusting for ordered assessment time point); *baseline outcome score* (adjusted for initial clinical severity); and *smart‐messaging* (the target predictor comparing smart‐messaging versus no‐messaging)*.* Analysis was conducted using SPSS 24.

Ethical approval was obtained from the National Research Ethics Service, London‐Riverside Committee (reference 14/LO/1102).

## Results

There were no significant differences identified between the smart‐messaging and no‐messaging groups on any observed baseline clinical or demographic characteristics (Table 3). There were also no significant differences between smart‐messaging users and non‐users at 3‐month follow‐up on any outcome (all *Z* < 1.75, *p *> .080).

**Table 3:** Participant characteristics

	Post‐treatment messaging *n* = 15	No messaging *n* = 38
Demographics		
Females	12 (80%)	30 (79%)
Mean age (*SD*)	39 (15)	37 (17)
Ethnicity		
White British	10 (67%)	29 (76%)
Others	5 (33%)	9 (24%)
Employed	6 (40%)	12 (32%)
Clinical characteristics		
Baseline SHAI (*SD*)	26.7 (4.8)	27.0 (5.2)
Baseline PHQ‐9 (*SD*)	13.2 (6.6)	13.2 (6.5)
Baseline GAD‐7 (*SD*)	13.7 (5.7)	12.5 (5.9)
Baseline PHQ‐15 (*SD*)	11.7 (5.9)	15.0 (4.8)
Baseline WSAS (*SD*)	18.9 (11.3)	20.5 (11.5)
Baseline EQ‐5D‐5L Utility Index (*SD*)	0.609 (.229)	0.627 (.268)
Baseline VAS (*SD*)	54.3 (22.4)	54.0 (20.4)
Mean number of SCID Diagnoses (Range)	6 (0‐14)	7 (1‐16)
Generalized anxiety disorder	12 (80%)	24 (63%)
Hypochondriasis	9 (60%)	22 (58%)
Somatoform disorder	8 (53%)	25 (66%)
Current depressive episode	8 (53%)	26 (68%)
Panic disorder	10 (67%)	23 (61%)
Mean chronic physical health problems	1 (0–6)	1 (0–4)
Median sessions attended (IQR)	12 (4)	12 (4)

Note

Abbreviations: EQ‐5D‐5L, EuroQol – 5 ‐Dimensions – 5 ‐Levels; GAD‐7, Generalised Anxiety Disorder – 7 ‐items; IQR, interquartile range; PHQ‐15, Patient Health Questionnaire – 15 ‐items; PHQ‐9, Patient Health Questionnaire – 9 ‐items; SCID, Structured Clinical Interview for the Diagnostic and Statistical Manual of Mental Disorders IV; SD, standard deviation; SHAI, Short form Health Anxiety Inventory; VAS, visual analogue scale; WSAS, Work and Social Adjustment Scale.

**Figure 1:**
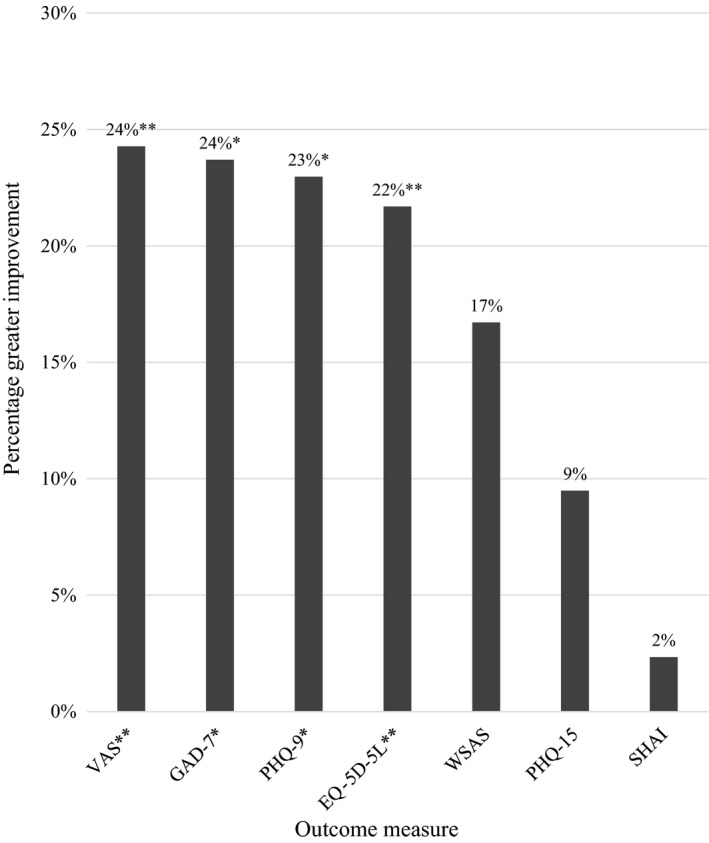
Percentage greater outcome improvement of post‐CBT smart‐messaging users versus non‐users. *Note*. EQ‐5D‐5L, EuroQol – 5 Dimensions – 5 Levels; GAD‐7, Generalised Anxiety Disorder – 7 items; PHQ‐15, Patient Health Questionnaire – 15 items; PHQ‐9, Patient Health Questionnaire – 9 items; SHAI, Short Health Anxiety Inventory; VAS, visual analogue scale; WSAS, Work and Social Adjustment Scale. **p <. 01; *p < .05.

Smart‐messaging users responded to an average 13 messages (54%; *SD* = 5.7; range 2‐24) of the 24 response opportunities. There were trends for greater outcome improvements among smart‐message users compared to non‐users on all measures across 12‐month follow‐up when controlling for baseline differences in severity and change over time (Figure 1). Differences were significant for overall health (VAS; *B* 2All beta values reported are non‐standardized.
* = *13.14*, SE B = *4.40, *p* = .004), generalized anxiety (GAD‐7; *B* = −3.04*, SE B = 1.18, p* = .014), depression (PHQ‐9; *B* = −3.03*, SE B = *1.35*, p* = .029), and quality of life (EQ‐5D‐5L; *B* = .135*, SE B* = .048*, p* = .008); but non‐significant for work and social adjustment (WSAS; *B* = −3.35*, SE B = *2.21*, p* = .137), somatic symptoms (PHQ‐15; *B* = −1.33*, SE B = *1.20, *p* = .273), and health anxiety (SHAI; *B* = *−*0.63*, SE B = *1.75*, p* = .721). Visual inspection of mean outcome trends over time indicates that differences between smart‐message users and non‐users grew over the 12‐month follow‐up (see Supporting information for outcome trends between groups over time). The greater improvements reported by smart‐message users met the criteria for minimal clinically significant difference from non‐users in quality of life (EQ‐5D, 0.135), general health (VAS, 13.14), and generalized anxiety (GAD‐7, 3.04) (Pickard, Neary, & Cella, 2007; Walters & Brazier, 2005; Zahra *et al.*, 2014). Furthermore, a greater proportion of smart‐message users achieved minimal clinically significant improvement at 12‐month follow‐up than non‐users in general health (79 vs. 42%), generalized anxiety (79 vs. 56%), depression (57 vs. 40%), quality of life (35 vs. 25%), and health anxiety (92 vs. 81%) with similar proportions of clinically important change in somatic symptoms (21 vs. 19%).3No agreed minimal clinically important difference is currently available for the WSAS.


## Discussion

This study offers initial evidence that post‐treatment smart‐messaging may support greater outcome improvement over a 12‐month follow‐up period. The methods used suggest that participants receiving smart‐messaging may have better retained or continued to develop learning from CBT sessions several months after treatment ended. Engagement data indicate that participants responded to the majority of smart‐message prompts, suggesting there was active uptake of the intervention among users that may have contributed to observed differences.

The study must still be seen as initial evidence, given the small sample of participants who opted to use smart‐messaging. The low uptake also suggested that the acceptability of the intervention requires further investigation. Low uptake is common problem in digital health interventions (e.g., Gilbody *et al.*, 2015), partly due to the self‐directed uptake and engagement required. Therefore, the self‐selecting sample is an important limitation given that there was no control for patient self‐motivation factors. The lack of a standardized and clear means of presenting the smart‐messaging intervention may have contributed to inconsistent patient uptake: Information provision was variable, and some patients received limited understanding of the intervention’s potential for benefit. This may help explain the low uptake and suggests a clear standardized rationale be used in future research. Nonetheless, there were no identified differences between groups in clinical or demographic characteristics suggesting a relatively comparable sample. Furthermore, baseline severity and change over time was controlled in the main analysis to support a fair comparison between groups. However, the possibility remains that the self‐selecting sample of smart‐messaging users may differ from the remaining sample on some unassessed, but significant characteristic, such as motivation or differences in effects between therapists. Systematic data were not collected on the reasons for refusal among non‐users of the intervention. This would help clarify means of addressing engagement.

Despite consistent trends for greater improvement among smart‐message users on all outcomes, there was no significant difference between groups on the primary treatment target: health anxiety. If smart‐messaging was indeed enhancing therapeutic effects, this finding suggests that it lacked specificity, as the CBT intervention was particularly focused on improving health anxiety.

Overall, this study indicates that the use of smart‐messaging to improve relapse prevention is worthy of formal evaluation in a randomized controlled trial, due to its potential as a cheap and accessible means of enhancing CBT outcomes. Study 2 presents initial evidence from the application of smart‐messaging in routine clinical practice.

## STUDY 2: FEASIBILITY OF SMART‐MESSAGING USERS IN ROUTINE CARE

## Method

### Design

This study presents a feasibility clinical case series of participants who received CBT in a clinical psychology service for cancer participants experiencing anxiety and/or depression. Participants completing treatment were offered post‐treatment smart‐messaging, and their well‐being was tracked over the 25‐week smart‐messaging follow‐up period using the well‐being scores that they texted in responses. The trends of well‐being were then assessed for stability and consistency.

### Participants

Fourteen participants4Two patients using smart‐messaging in the case series were not diagnosed with cancer, but did *experience* anxiety and depression at baseline, so remain included. were referred by cancer care staff to a clinical psychology service and completed CBT offered by one of four therapists. Participants attended a mean of 12.4 sessions (*SD* = 5.0); they had a mean age of 53 (*SD* = 13.6), with primary cancers from five different sites (5 breast, 4 lower gastrointestinal, 1 haematology, 1 brain, and 1 head and neck). Four participants were receiving palliative care for metastatic cancer, and the remainder received curative treatment. All participants consented to the use of smart‐messaging, as previously described, and anonymous use of text data. The results of Study 1 were used to explain the method and potential benefits of smart‐messaging to patients recruited in Study 2.

### Interventions

#### CBT

The clinical psychology service offered participants CBT interventions with the aim of addressing problematic responses to cancer diagnosis and treatment rather than aiming to change symptoms of cancer themselves. For example, several participants included in the case series were highly anxious about cancer recurrence after treatment was complete, but reactions such as repeated body checking and reassurance‐seeking only served to maintain anxiety (Salkovskis, Warwick, & Deale, 2003). Unhelpful cognitive or behavioural reactions were identified and collaboratively reduced or stopped in a similar manner to CBT in the above‐described trial.

#### Smart‐messaging

The same set‐up and running procedures for smart‐messaging were used as in the above‐described trial.

### Procedure

Participant ratings of well‐being texted in response to smart‐messaging each week (0–5; where 0 is the worst and 5 is the best) were collated by the clinical psychology service during the 25‐week smart‐messaging follow‐up period. An identification key was used to link the anonymous unique identifier used by the smart‐messaging software with the participating participant.

### Outcome measures

Well‐being (0‐5) scores were used as a proxy outcome measure, and the stability of well‐being scores was used as an assessment of relapse. Therefore, where there was no significant change in well‐being score across follow‐up time, the gains achieved from therapy were deemed to be retained and relapse prevented. However, if there was a significant deterioration in well‐being score trends, it was considered a relapse.

### Method of analysis

Simulation modelling analysis (SMA; Borckardt *et al.*, 2008) was applied to give an individual‐level assessment of stability in weekly well‐being scores over the 25‐week smart‐messaging follow‐up period. SMA allows for examination of individual time series in terms of change over repeated‐assessment time points and their statistical significance using bootstrapping methods that account for the length and autocorrelation of observed data streams. This approach generates fewer Type I and Type II errors than visual data inspection (Borckardt *et al.*, 2008). Applied to well‐being scores over time, SMA computes a correlation (Pearson *r*) between the observed data and a linear slope vector for time: Such that negative coefficients indicate deterioration over time, positive coefficients indicate improvement, and zero coefficients indicate stability (no change). SMA then simulates 1,000 data streams, with the same autocorrelation and *n* as the observed data, drawn randomly from a null distribution of data streams. Finally, SMA produces a *p* value for the observed coefficient: representing the empirical probability of observing a coefficient of equal or greater magnitude in a null distribution of data streams (with matched properties, in terms of *n* and autocorrelation). Thus, non‐significant values indicate that an individual’s scores are stable over the follow‐up period. The purpose‐made SMA software was used to complete the analysis (available at http://www.clinicalresearcher.org/).

Overall stability in well‐being across cases over the smart‐messaging follow‐up period was assessed using Wilcoxon signed ranks test of (group level) difference between the first week well‐being score and the last recorded well‐being score (average week at 23).

Ethical approval was not required as Study 2 evaluated routinely collected data from clinical practice, but the evaluation was registered with the participating health care organization.

## Results

**Figure 2:**
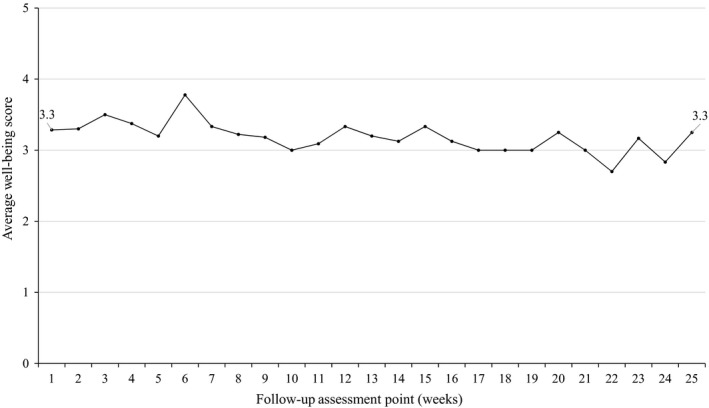
Average weekly well‐being score over 6‐month follow‐up.

Of the 14 cases assessed, 11 (79%) showed individual stability in well‐being scores and 3 (21%) showed deteriorating trends over the 6 months following CBT completion (Figure 2). Participants responded to the 25 message requests for well‐being scores an average of 17 times, giving a mean response rate of 68% (*SD* = 6; Range = 7–25).

At the group level, the first and last recorded well‐being scores had identical means (3.3, *SD* = 1.1). Given that last recorded well‐being scores were received on average at week 23, this suggests overall stability of well‐being in the 6 months after CBT was completed (*Z* = .07, *p* = .942).

## Discussion

This study suggests there is a high rate of stability in well‐being ratings reported in the 6 months following CBT by users of personalized smart‐messaging. Furthermore, there is a high rate of engagement with the smart‐messaging intervention, with participants responding to smart‐message requests on the majority of occasions.

If the use of idiosyncratic well‐being ratings is accepted as a proxy assessment of outcome stability, rates of relapse in this study compare favourably with other studies of CBT follow‐up in routine care without smart‐messaging (Delgadillo *et al.*, 2018). However, this study cannot offer a direct comparison with participants who did not use smart‐messaging and it is unclear whether 0–5 well‐being scores offer a meaningful assessment of outcome stability. Future research could include concurrent standardized symptom assessments alongside the 0‐to‐5 well‐being scale to assess its reliability and concurrent validity. If the method used is found to have adequate sensitivity, reliability, and validity, it could offer a simple and effective means of follow‐up outcome assessment.

In summary, this study suggests that smart‐messaging may be a feasible and engaging tool for users after CBT completion in routine care.

## GENERAL DISCUSSION

This paper offers evidence that using personalized smart‐messaging, tailored to patients’ emotional state, may reduce relapse after CBT. Study 1 suggests that smart‐messaging may contribute to greater improvement in a range of clinical outcomes. Study 2 indicates feasibility of smart‐messaging in routine care, due to a high level of patient engagement with the intervention in routine care and potential use of smart‐messaging to track post‐treatment progress in clinical practice.

The studies reported support existing evidence that smart‐messaging, through a brief and low‐intensity intervention, might have a positive impact on physical and mental health (Rathbone & Prescott, 2017). This paper also supports current evidence on the effectiveness of digital methods for relapse prevention in anxiety and depressive disorders (Hennemann *et al.*, 2018). This paper adds to the literature by providing evidence that digital relapse prevention methods can be fully integrated with traditional, therapist‐facilitated CBT (even if delivered over videoconferencing). This paper also extends evidence for smart‐messaging by showing the potential benefits of personalizing the messages patients received in line with key therapeutic learning. Current CBT theory may support a mechanistic explanation of the results reported: The learning of key CBT competencies enhances post‐treatment prognosis (Strunk *et al.*, 2007); it is possible that smart‐messaging enhances and potentially continues this learning beyond the end of therapy sessions.

Existing digital health interventions have indicated the potential to increase reach and accessibility of relapse prevention alongside improvements in cost‐effectiveness. This study suggests that integration of smart‐messaging can add greater long‐term benefits to relatively brief CBT with no additional therapist time required. Therefore, this study offers initial indications for an intervention that can be spread easily and may save clinical resources alongside potential for improved patient benefit.

The use of multiple independently collected physical health, mental health, and functional outcomes over a 12‐month follow‐up period meant that outcomes could be compared in a number of domains. Within this design, the consistently superior performance among smart‐messaging users gave a persuasive case for the potential value of the intervention. The illustration of smart‐messaging’s use in routine practice helped demonstrate the pragmatic feasibility of the intervention through data on usage and stability of well‐being.

However, this paper remains a proof‐of‐concept study requiring more rigorous assessment in future research, given the limitations associated with the use of a self‐selecting sample in Study 1 and the lack of a comparator group or validated outcomes in Study 2. Longer follow‐up periods would also help to give a clearer indication of any relapse prevention benefits offered by smart‐messaging. Although relapse often occurs within the first year after CBT, 2‐year follow‐up gives a better indication of long‐term prognosis (e.g., Kuyken *et al.*, 2008). The addition of a systematic evidence‐based rationale for the use of smart‐messaging in Study 2, which was missing from Study 1, may explain the low uptake observed. This could help improve engagement in future research.

In future research, smart‐messaging should be formally evaluated in a randomized controlled trial with a long post‐CBT follow‐up period. Future research could also evaluate whether personalization of messages has an added benefit over a standardized post‐treatment messaging protocol. This type of research would more clearly give evidence for the feasibility and acceptability of the intervention, which remains unclear given the low uptake in Study 1. Therefore, further testing may be required prior to a full trial. Future testing of post‐intervention smart‐messaging interventions would be strengthened by expanding the scope to examine its utility with different disorders, interventions, and patient populations. The mechanisms for any change associated with smart‐messaging should also be investigated in future research. Established methods of assessing CBT competencies could be used to assess whether these competencies are maintained or enhanced by smart‐messaging.

In summary, patients who receive CBT for anxiety or depressive disorders may benefit from personalized post‐treatment smart‐messaging. However, larger‐scale randomized evaluation is required before a dependable assessment of its value can be reached.

## Conflicts of interest

All authors declare no conflict of interest.

## Author contributions

Sam Malins, Sanchia Biswas (Conceptualization; Investigation; Writing – original draft; Writing – review & editing) Shireen Patel (Data curation; Investigation; Project administration; Resources; Writing – original draft; Writing – review & editing) Jo Levene (Conceptualization; Investigation; Supervision; Writing – original draft; Writing – review & editing) Nima Moghaddam (Formal analysis; Methodology; Software; Writing – original draft; Writing – review & editing) Richard Morriss (Conceptualization; Funding acquisition; Investigation; Supervision; Writing – original draft; Writing – review & editing).

## Supporting information


**Figure S1** **.** Change in general health (VAS) over time between smart‐messaging users and non‐users (higher scores indicate greater health improvement).
**Figure S2** **. **Change in generalized anxiety (GAD‐7) over time between smart‐messaging users and non‐users (lower scores indicate greater symptom improvement).
**Figure S3** **.** Change in depression (PHQ‐9) over time between smart‐messaging users and non‐users (lower scores indicate greater symptom improvement).
**Figure S4** **.** Change in quality of life (EQ‐5D‐5L) over time between smart‐messaging users and non‐users (higher scores indicate greater quality of life improvement).
**Figure S6** **.** Change in somatic symptoms (PHQ‐15) over time between smart‐messaging users and non‐users (lower scores indicate greater symptom improvement).
**Figure S7** **.** Change in health anxiety (SHAI) over time between smart‐messaging users and non‐users (lower scores indicate greater symptom improvement).

## Data Availability

The data that support the findings of this study are openly available on figshare at https://doi.org/10.6084/m9.figshare.9976208.v1.
