# Supplementary material for: Preventing relapse with personalized smart‐messaging after cognitive behavioural therapy: A proof‐of‐concept evaluation
Source: Br J Clin Psychol. 2020 Jan 20;59(2):241–59. doi: 10.1111/bjc.12244 (PMC7216897; doi:10.1111/bjc.12244)
Supplement: Supplementary file 1 — Figure S1 . Change in general health (VAS) over time between smart‐messaging users and non‐users (higher scores indicate greater health improvement). Figure S2 . Change in generalized anxiety (GAD‐7) over time between smart‐messaging users and non‐users (lower scores indicate greater symptom improvement). Figure S3 . Change in depression (PHQ‐9) over time between smart‐messaging users and non‐users (lower scores indicate greater symptom improvement). Figure S4 . Change in quality of life (EQ‐5D‐5L) over time between smart‐messaging users and non‐users (higher scores indicate greater quality of life improvement). Figure S6 . Change in somatic symptoms (PHQ‐15) over time between smart‐messaging users and non‐users (lower scores indicate greater symptom improvement). Figure S7 . Change in health anxiety (SHAI) over time between smart‐messaging users and non‐users (lower scores indicate greater symptom improvement). [file BJC-59-241-s001.docx]

*Supplementary Figure 1.* Change in general health (VAS) over time between smart-messaging users and non-users (higher scores indicate greater health improvement)

*Supplementary Figure 2.* Change in generalized anxiety (GAD-7) over time between smart-messaging users and non-users (lower scores indicate greater symptom improvement)

*Supplementary Figure 3.* Change in depression (PHQ-9) over time between smart-messaging users and non-users (lower scores indicate greater symptom improvement)

*Supplementary Figure 4.* Change in quality of life (EQ-5D-5L) over time between smart-messaging users and non-users (higher scores indicate greater quality of life improvement)

*Supplementary Figure 5.* Change in work and social impairment (WSAS) over time between smart-messaging users and non-users (lower scores indicate greater functional improvement)

*Supplementary Figure 6.* Change in somatic symptoms (PHQ-15) over time between smart-messaging users and non-users (lower scores indicate greater symptom improvement)

*Supplementary Figure 7.* Change in health anxiety (SHAI) over time between smart-messaging users and non-users (lower scores indicate greater symptom improvement)
